# The Assessment of 3D Digital Models Using GOSLON Yardstick Index: Exploring Confounding Factors Responsible for Unfavourable Treatment Outcome in Multi-Population Children With UCLP

**DOI:** 10.3389/fped.2021.646830

**Published:** 2021-06-28

**Authors:** Sanjida Haque, Mohd Fadhli Khamis, Mohammad Khursheed Alam, Amir Wan Muhamad Wan Ahmad

**Affiliations:** ^1^Orthodontic Unit, School of Dental Sciences, Universiti Sains Malaysia, Kota Bharu, Malaysia; ^2^Oral Biology and Forensic Odontology Unit, School of Dental Sciences, Universiti Sains Malaysia, Kota Bharu, Malaysia; ^3^Forensic Odontology Unit, Hospital Universiti Sains Malaysia, Kota Bharu, Malaysia; ^4^Orthodontic Department, College of Dentistry, Jouf University, Sakaka, Saudi Arabia; ^5^Biostatistics Unit, School of Dental Sciences, Universiti Sains Malaysia, Kota Bharu, Malaysia

**Keywords:** UCLP, multi populations, treatment outcome, 3D digital models, GOSLON Yardstick

## Abstract

To evaluate dental arch relationship (DAR) using GOSLON Yardstick and also to explore the association between multiple factors (age, gender, UCLP types, UCLP side, Family history of cleft, family history of Class III malocclusion, techniques of cheiloplasty, techniques of palatoplasty) and DAR in children unilateral cleft lip and palate (UCLP) in different populations. Two hundred fifty-five laser scanned 3D digital models (LS3DM) of UCLP children (5–12 years) from Malaysia, Bangladesh, and Pakistan were included. The intra- and inter-examiner agreements were evaluated by kappa statistics, to compare the GOSLON mean score between the populations and to explore the responsible factors that affect DAR, one way ANOVA, and crude logistic regression analysis was used, respectively. The mean GOSLON score was 2.97; 3.40 and 3.09 in Malaysia, Bangladesh, and Pakistan, respectively. Twenty seven, 40, and 30 subjects were in unfavourable (category rating 4 and 5) groups in Malaysia, Bangladesh, and Pakistan, respectively. A significant association was found between techniques of palatoplasty (*p* = 0.03; *p* = 0.04 and *p* = 0.04 in Malaysia, Bangladesh, and Pakistan, respectively) and unfavourable DAR. Different cheiloplasty techniques (*p* = 0.04) and gender (*p* = 0.03) also exhibited noteworthy associations with unfavourable DAR in the Bangladeshi population. Bardach techniques of palatoplasty were significantly associated with unfavourable DAR in all three populations. Moreover, male UCLP and modified Millard techniques of cheiloplasty were also associated with unfavourable DAR in the Bangladeshi population.

## Introduction

The prevalence of unilateral cleft lip and palate (UCLP) varies between countries with higher rates have been reported in Asians and American Indians which is 1 per 500 births whereas African derivative populations have the lower rates (1 per 2,500 births) (1). Being Asian country, Malaysia, Bangladesh and Pakistan also some extent of high rate of prevalence of cleft lip and palate. In Malaysia, it is found 1 per 941 live births (2) and 1 per 523 live births are reported in Pakistan (3) whereas 3.9:1000 live births are reported in Bangladesh (4) which is relatively higher than other regions in Asia.

Despite variations between these countries, the impact of cleft lip and palate on both aesthetic and functional malformations are equally observed since birth (1, 5). The treatment of UCLP patients is multifaceted, prolonged, and complicated. A series of surgeries is aimed at the treatment of this congenital anomaly for the correction of esthetic and functional development. The manifestation of undeveloped maxillary growth and occurrence of Class III malocclusion is frequent in UCLP patients (6, 7). The growth and development of the maxilla are affected by different techniques of primary lip and palate surgeries. Not only surgeries but also genotype factors have influences on the growth of maxilla reported previously (8, 9).

In this contemporary era of clinical practise, the evaluation of treatment outcomes with reliable and sound evidence is crucial after the early management of UCLP patients. This evaluation provides the suggestion of appropriate orthodontic treatment strategy and surgical methods for the primary repair of CLP thus the entire treatment process could be more successful. The treatment outcome can be evaluated by assessing the dental arch relationship (DAR), craniofacial morphology, maxillary arch dimension, etc. DAR is one of the most ideal measuring tools that can give a complete idea about facial growth and occlusion as well.

Many indices have been established to assess the DAR. Among them, the GOSLON (Great Ormond Street, London, and Oslo) Yardstick (GY) (10), is the most extensively used and relevant index for this determination of evaluating the DAR (8, 9, 11–17). However, only few studies have evaluated both the DAR and effects of multiple factors on the DAR together (8, 9, 12, 13, 17) and the majority of these studies was using plaster dental casts.

Based on the literature search there were few studies done by using 3D digital models (14–16). Thus far, to the best of the author's knowledge, no reported data to date was found on Malaysian, Bangladeshi and Pakistani populations that evaluate the multiple factors (age, gender, family history of cleft, UCLP type and side, techniques of cheiloplasty and palatoplasty) which may influence the treatment outcome using a 3D digital model. Therefore, the present study attempted to evaluate DAR using GY and also to explore the association between multiple factors and DAR of Malaysian, Bangladeshi, and Pakistani UCLP subjects.

## Materials and Methods

A total of 255 pretreatments orthodontic plaster dental casts of UCLP (involved only unilateral lip and full palate cleft) children from three different populations were selected into this study which consisted of 85 subjects in each population. The dental casts were collected during subjects' first visit to the orthodontist in a renowned hospital separately in Malaysia, Bangladesh, and Pakistan between 2010 and 2013. The subjects' age ranged between 5 and 12 years and who had completed their cheiloplasty and palatoplasty and without bone grafting were included in this study whereas any kind of missing records, syndromic UCLP, bilateral cleft, isolated cleft lip, and cleft palate were not included for further assessment. Those who fulfilled the inclusion and exclusion criteria were selected by a simple random sampling method.

**Figure 1:**
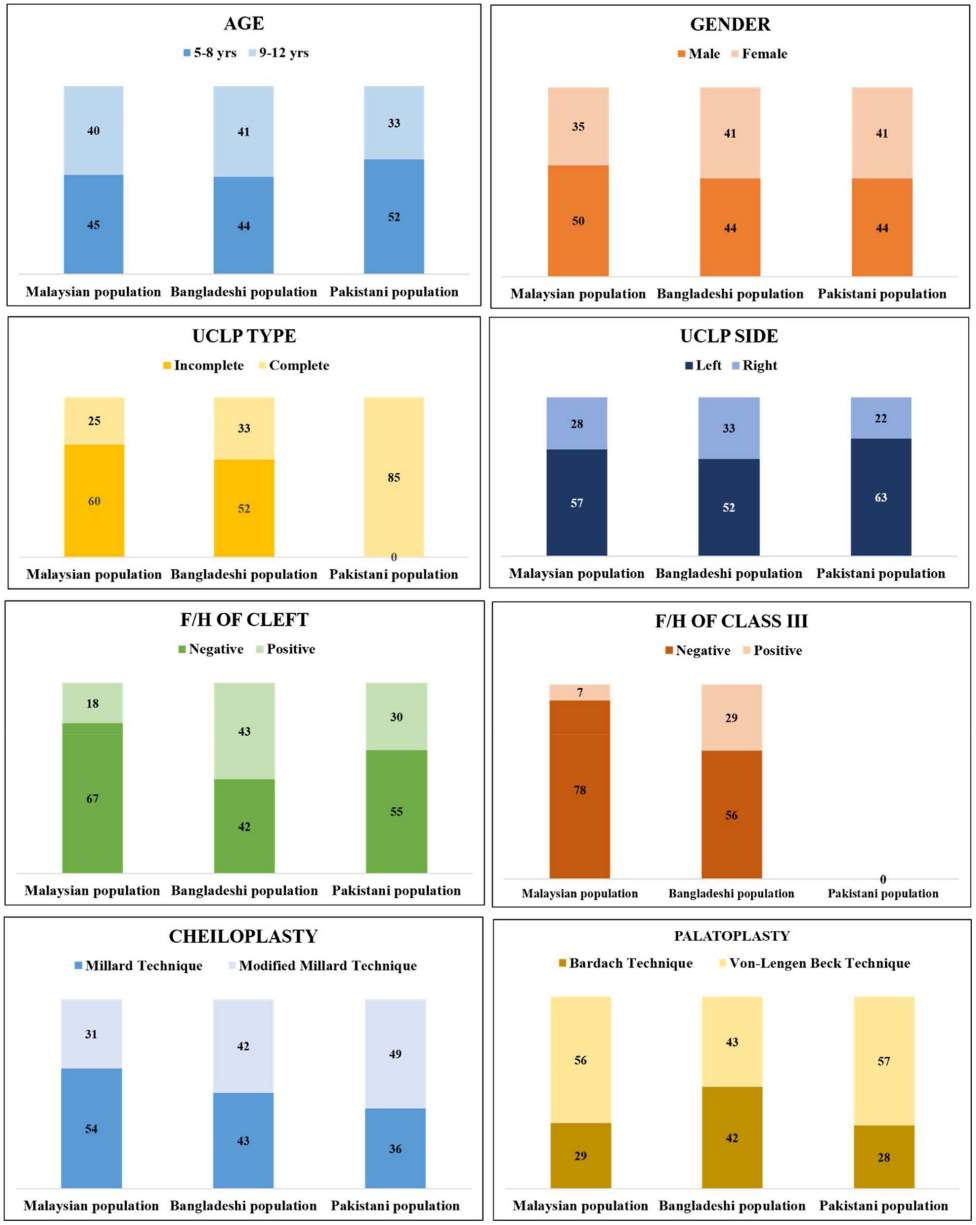
Distribution of all subjects from three populations with multiple factors. (All the Pakistani UCLP subjects were complete type of UCLP. No record was found regarding family history of class III malocclusion in Pakistani UCLP subjects).

The sample size was calculated on the bases of a ratio of 1 predictor: 20 cases. A total of eight predictors were presented in this prime study. Hence, 160 was the minimum projected sample size for all populations together. Ensuing the inclusion and exclusion criteria, as a final point, 255 samples have been selected for this study. The distribution of all subjects from three populations with multiple factors was shown in Figure 1.

**Figure 2:**
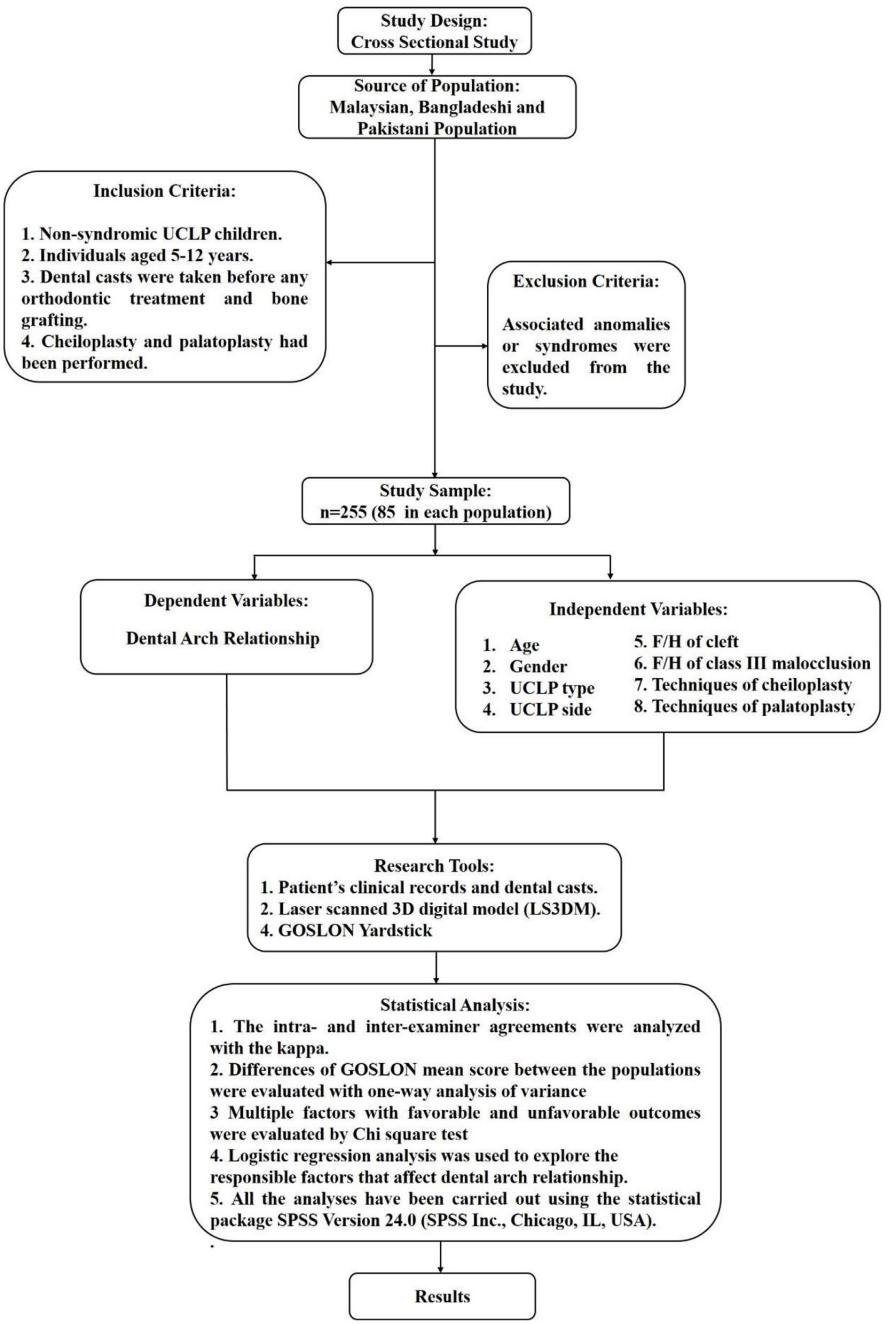
The flow chart of the study.

The demographic information of the subjects, selection criteria, dependent and independent variables, and statistical analyses detail was given in a flowchart (Figure 2).

**Figure 3:**
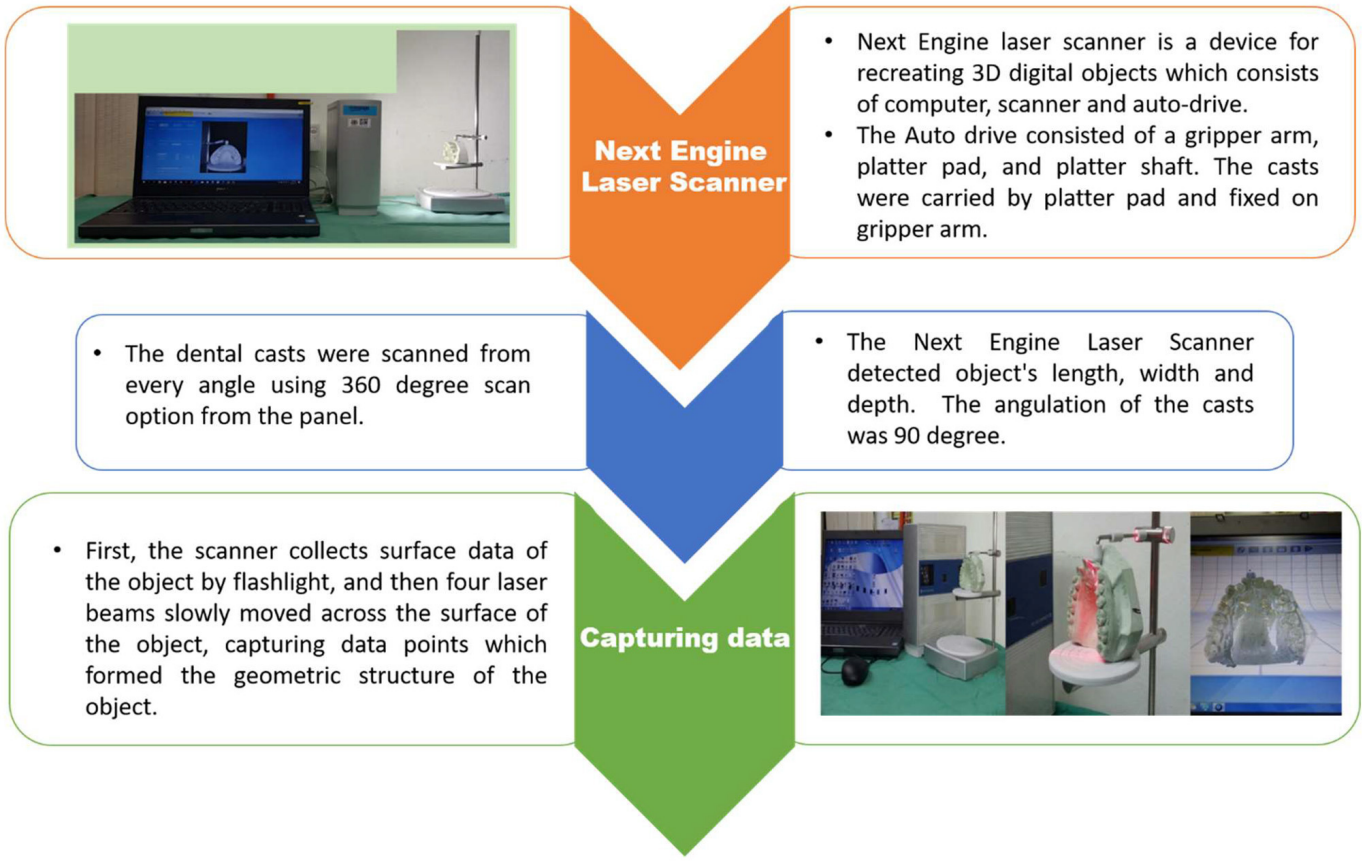
Conversion of the dental casts into LS3DM.

**Figure 4:**
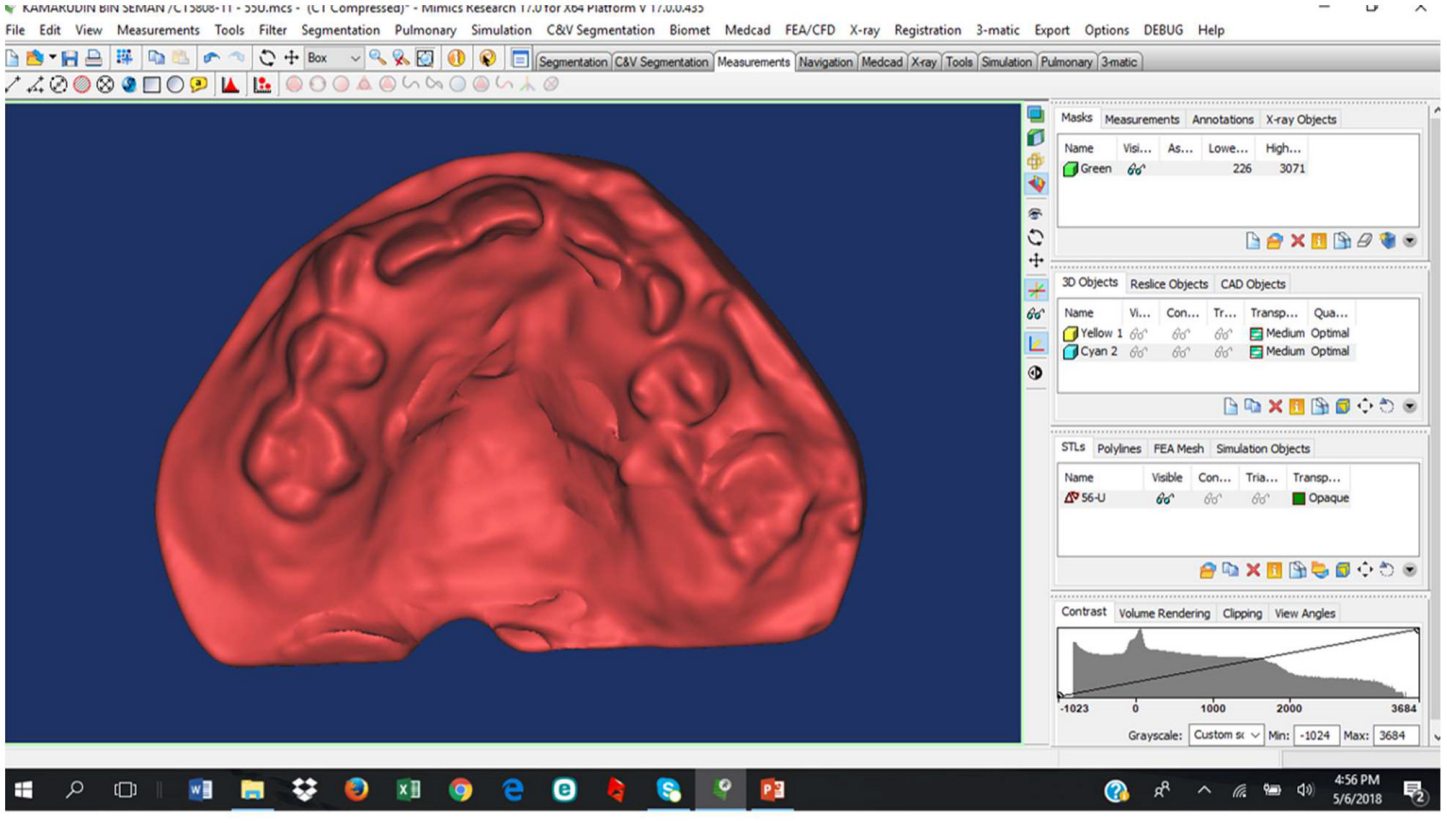
LS3DM in the STL format.

All the 255 dental casts (both upper and lower jaws) were scanned and converted into LS3DM using the Next Engine laser scanner (Santa Monica) by an experienced craniofacial lab technician following the standard method of scanning of the Next engine laser scanner (Figure 3). All scanned data coordinates (in x, y, z) were transferred into STL format (Figure 4) and finally, DAR was evaluated using GY index.

Five categories are assessed in GY; 1: excellent; 2: good; 3: fair; 4: poor; 5: very poor which provided the dentofacial growth and development, and also discovered the differences of DAR. Group 1 reflects the excellent treatment outcome which presents a favourable relationship, advantageous skeletal form, a positive overjet, and overbite with the presentation of Angle Class II division 1 malocclusion. Straightforward orthodontic treatment or no treatment is required in this group. Group 2 reflects the good treatment outcome which also shows a favourable relationship with the Class I dental relationship. Straightforward orthodontic treatment is required in this group. Group 3 represents the fair treatment outcome with an edge-to-edge dental relationship. More complex orthodontic treatment is needed in this group. Group 4 reflects the poor treatment outcome presenting an unfavourable facial growth. Reverse overjet of 3–5 mm also shows in this group which point to the confines of orthodontic treatment, where the orthognathic surgery might be required in some cases. Group 5 represents the very poor treatment outcome with an extensive skeletal class III relationship with the need for compulsory surgical treatment.

For further analyses, the groups were collapsed into two groups i.e., favourable and unfavourable groups. Groups 1–3 were in favourable groups and groups 4 and 5 were in the unfavourable group. Subjects in the favourable group may need conventional orthodontics treatment whereas surgical treatment is required with subjects in the unfavourable groups.

**Figure 5:**
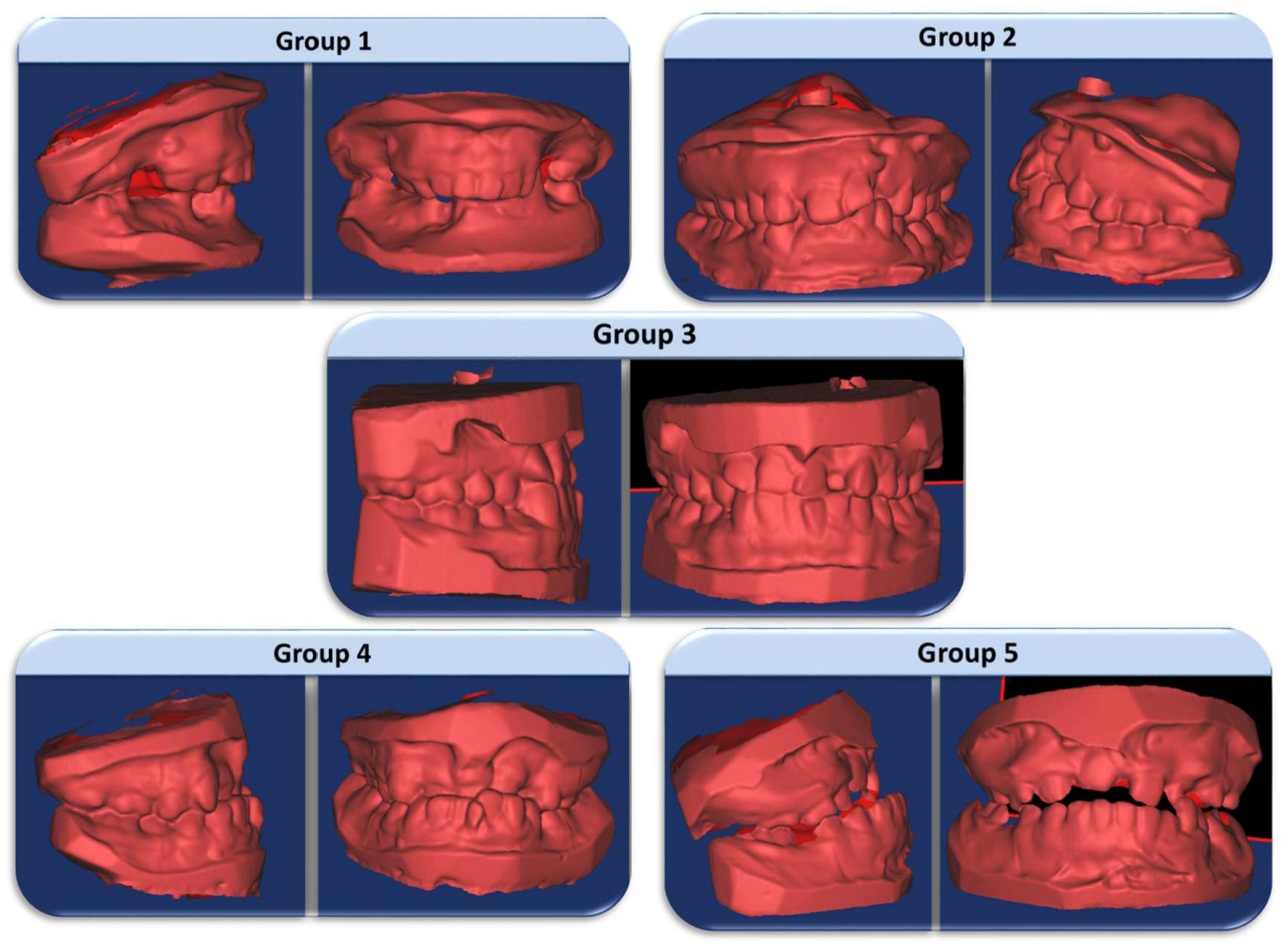
Five groups of GY model picture in 3D format.

Figure 5 shows five groups of GY model picture in 3D format.

Each population data collected with proper clearance and permission. Ethical consent has been obtained for specific population as approved by the USM Ethics Committee [USM/JEPeM/17100564].

## Results

### Reliability of GY

The kappa score was 0.70 and 0.63 for intra- and inter-examiner agreements, respectively for GY which recommended good agreements.

### Assessment of Treatment Outcome Based on GOSLON Score

Table 1 shows the score distribution and the mean GOSLON score of UCLP subjects of three populations using GY.

**Table 1:** The score distribution and the mean GOSLON score of UCLP subjects of three populations using GY.

	**Malaysia (*n*)**	**Bangladesh (*n*)**	**Pakistan (*n*)**
Group 1	4	3	4
Group 2	17	11	25
Group 3	44	31	26
Group 4	17	29	19
Group 5	3	11	11
Mean GOSLON Score	2.97	3.40	3.09

### Comparison of the Mean GOSLON score Between Three Populations

Table 2 shows mean GOSLON scores for the three populations were compared with ANOVA and followed by *post hoc* Tamhane tests for pairwise comparisons. The only statistically significant difference was between the Malaysian and Bangladeshi populations (*p* = 0.01). Tamhane pairwise tests found that Bangladesh has a higher GY score than Malaysian. Tamhane test was selected due to non-homogenous variance tests (Levene's Test).

**Table 2:** Comparison of mean GOSLON scores between the three populations.

**Population**	**Mean GOSLON score**	**SD**	**Inter-population differences (for *p* < 0.05)**
Malaysia	2.97	0.86	Mal vs. Ban
Bangladesh	3.40	1.00	Ban vs. Mal
Pakistan	3.09	1.10	

*Mal, Malaysia; Ban, Bangladesh; SD, standard deviation*.

### Comparison of Factors Between Favourable and Unfavourable Groups

#### Malaysian Population

Fifty eight and 27 subjects remained in the favourable and unfavourable groups respectively.

#### Bangladeshi Population

Forty five and 40 subjects reported in favourable and unfavourable groups respectively.

#### Pakistani Population

Fifty five and 30 subjects remained in favourable and unfavourable groups respectively.

The distribution of the subjects of three populations along with multiple factors using GY are shown in Table 3.

**Table 3:** Distribution of subjects with multiple factors in favourable and unfavourable groups using GY in Malaysian, Bangladeshi and Pakistani UCLP children.

**Variable**	**Tx outcome of Malaysian population**, ***n*** **(%)**		**Tx outcome of Bangladeshi population**, ***n*** **(%)**		**Tx outcome of Pakistani population**, ***n*** **(%)**	
	**Favourable**	**Unfavourable**	**Favourable**	**Unfavuorable**	**Favourable**	**Unfavuorable**
**Age**						
5–8 years	29 (34.1)	16 (18.8)	21(24.7)	23 (27.1)	36 (42.2)	16 (18.8)
9–12 years	29 (34.1)	11 (12.9)	24 (28.2)	17 (20.0)	19 (22.4)	14 (16.5)
**Gender**						
Male	38 (44.7)	12 (14.1)	19 (22.4)	25 (29.4)	24 (28.2)	20 (23.5)
Female	20 (23.5)	15 (17.6)	26 (30.6)	15(17.6)	31 (36.5)	10 (11.8)
**UCLP Side**						
Left	40 (47.1)	17 (20.0)	30 (35.3)	22 (25.9)	43 (50.6)	20 (23.5)
Right	18 (21.2)	10 (11.8)	15 (17.6)	18 (21.2)	12 (23.5)	10 (11.8)
**UCLP type**						
Incomplete	46 (54.1)	14 (16.5)	29 (34.1)	23 (27.1)	N/A^*^	N/A^*^
Complete	12 (14.1)	13 (15.3)	16 (18.8)	17 (20.0)		
**F/H of Cleft**						
Negative	44 (51.8)	23 (27.1)	24 (28.2)	18 (21.2)	37 (43.5)	18 (21.2)
Positive	14 (16.5)	4 (4.7)	21 (24.7)	22 (25.9)	18 (21.2)	12 (14.1)
**F/H of Class III**						
Negative	54 (63.5)	24 (28.2)	32 (37.6)	24 (28.2)	N/A^**^	N/A^**^
Positive	4 (4.7)	3 (3.5)	13 (15.3)	16 (18.8)		
**Cheiloplasty**						
MT	41 (48.2)	13 (15.3)	17 (20.0)	26 (30.6)	23 (27.1)	13 (15.3)
MMT	17 (20.0)	14 (16.5)	28 (32.9)	14 (16.5)	32 (37.6)	17 (20.0)
**Palatoplasty**						
BT	14 (16.5)	15 (17.6)	18 (21.2)	25 (29.4)	13 (15.3)	15 (17.6)
VLT	44 (51.8)	12 (14.1)	27 (31.5)	15 (17.6)	42 (49.4)	15 (17.6)

*UCLP, unilateral cleft lip and palate; F/H Cleft, Family history of cleft; F/H C-III, Family history of class III malocclusion; MT, Millard technique; MMT, modified Millard technique; BT, Bardach Technique; VLT, Von-Lengenbeck Technique*.

* *all the Pakistani UCLP subjects were complete type of UCLP*.

** *no record was found regarding family history of class III malocclusion in Pakistani UCLP subjects*.

### Treatment Outcome Associated With Multiple Factors (Favourable vs. Unfavourable DAR)

The crude logistic regression analysis was performed where *p*-value was set as <0.05 to consider of having significant association with DAR.

Techniques of palatoplasty (*p* = 0.03) was significantly associated with DAR in Malaysian population where subjects with Bardach technique of palatoplasty had 3.42 times the odds of unfavourable DAR compared to von Langenbeck technique.

In Bangladeshi population, gender (*p* = 0.03), techniques of cheiloplasty (*p* = 0.04) techniques of palatoplasty (*p* = 0.04) were significantly associated with DAR. Male subjects showed 2.93 times the odds ratio to unfavourable DAR than female subjects. The subjects who underwent with the modified Millard technique of cheiloplasty and Bardach technique of palatoplasty had 2.99 and 2.80 times, respectively, the odds to unfavourable DAR compared to the subjects with Millard technique of cheiloplasty and von Langenbeck technique of palatoplasty.

In Pakistani population, techniques of palatoplasty (*p* = 0.04) was significantly associated with DAR where subjects with Bardach technique of palatoplasty had 2.86 times the odds to unfavourable DAR in comparison with von Langenbeck technique.

Table 4 shows a brief result of association of multiple factors on treatment outcome of three different populations.

**Table 4:** Logistic regression analysis of multiple factors with treatment outcome (Favourable vs. unfavourable group) using GY in three population.

**Independent variable**	**Exp (B)**	**95% CI**		***p*-value**
		**Lower**	**Upper**	
**Malaysian population**				
Age	1.34	0.43	4.19	0.61
Gender	0.59	0.20	1.71	0.33
UCLP Side	0.53	0.16	1.73	0.30
UCLP Type	0.39	0.12	1.25	0.11
F/H of Cleft	2.03	0.44	9.41	0.37
F/H of C-III	0.42	0.07	2.38	0.32
Cheiloplasty	0.04	0.13	1.23	0.11
Palatoplasty	3.42	1.09	10.78	0.03^*^
**Bangladeshi population**				
Age	1.29	0.47	3.52	0.62
Gender	2.93	1.09	7.85	0.03^*^
UCLP Side	0.44	0.015	1.32	0.15
UCLP Type	1.15	0.37	3.60	0.81
F/H of Cleft	1.05	0.37	2.96	0.93
F/H of C-III	0.60	0.21	1.76	0.36
Cheiloplasty	2.99	1.07	8.38	0.04^*^
Palatoplasty	2.80	0.47	7.80	0.04^*^
**Pakistani population**				
Age	0.55	0.21	1.47	0.23
Gender	2.31	0.86	6.17	0.09
UCLP Side	0.75	0.25	2.24	0.60
F/H of Cleft	0.72	0.26	1.99	0.52
Cheiloplasty	0.90	0.33	2.44	0.84
Palatoplasty	2.86	1.05	7.76	0.04^*^

^*^F/H, family history.

* *p > 0.05*.

## DISCUSSION

In this present study, the authors evaluated and compared the DAR of UCLP subjects of 5–12 years in three different populations from Asia. Furthermore, the association of multiple factors with favourable and unfavourable DAR between the populations were also explored.

This range of age was chosen as most UCLP patients first received orthodontic treatment at the age of 5–6 years old (18). These UCLP subjects exhibited Class III malocclusions and other dental anomalies, and have yet to undergo alveolar bone grafting by the age of 12 (18). So the selection of this age range may represent the actual knowledge of treatment outcome to the orthodontist as well as to the surgeon. Same age range also documented in some other previous published studies (9, 17, 19).

In this study, Millard technique or modified Millard technique of cheiloplasty was the treatment of choice which was taken place at the age of 3–6 months and correspondingly two different surgical protocols of palatoplasty; Bardach technique or von Langenbeck technique was chosen for the subjects at 12–18 months of age. One skilled, qualified and experienced surgeon from each population performed all the surgeries of three populations.

We assessed 255 LS3DM of non-syndromic UCLP subjects from three populations using GY. The index was found to have good inter- and intra-examiner reliability in the present study which also corresponds with the findings of the earlier studies (20–22).

### Treatment Outcome

The mean GOSLON score of Malaysian, Bangladeshi, and Pakistani UCLP subjects was 2.97, 3.40, and 3.01, respectively. In the present study, the treatment outcome of Malaysian subjects was good to fair (between groups 2 and 3), representing 71.76% of all cases. Of the leftovers, 4.70% was excellent and 20% was poor and 3.5% was a very poor outcome. Two studies were conducted in Malaysia previously (19, 20). Zreaqat et al. (23) evaluated the treatment outcome among 82 UCLP subjects between 2004 and 2010 and reported a mean GOSLON score of 3.15 with 62% of all cases. On the other hand, 107 UCLP subjects were evaluated by Asif et al. (24) between 2000 and 2012; interestingly this study also found the same mean GOSLON score of 3.15 represented with 68% of all cases. The same GOSLON score of previous studies indicated a similar treatment outcome which could be due to the use of the same surgical technique/protocol. Both of the studies used plaster dental casts in their research.

The mean GOSLON score of Bangladeshi UCLP was 3.40. The treatment outcome of Bangladeshi subjects was fair to poor outcome representing 70.59% of all subjects. Only one study reported a mean GOSLON score of 3.238 with 68% of all subjects (25) for DAR in the Bangladeshi UCLP subjects using plaster dental casts.

The mean GOSLON score of Pakistani UCLP was 3.01. The treatment outcome of most Pakistani subjects of our study was fair; representing 30.59% of all cases. The remaining subjects were as follows 4.70% was excellent, 29.41% was good, 22.35% was poor and 12.94% was a very poor outcome. The current findings are consistent with the results of Arshad et al. (12); which is the only study found in the literature on the Pakistani population.

All these previous studies in Malaysia, Bangladesh, and Pakistan were done using plaster dental casts separately. Taking the advantage of the 3D digital model (26, 27) our study evaluated DAR in multi-population UCLP subjects where the results were comparable with those evaluation performed on plaster dental casts.

There have been many studies done about UCLP with GY in other populations. Different populations showed different results. For example, a recent multicenter study, found the mean GOSLON score ranged from 2.58 to 3.07 among three centres. They also reported one stage palatoplasty showed a low GOSLON score (better outcome) than two-stage palatoplasty (28). Another multicenter study reported a range of 3.16–3.70 mean GOSLON scores among different cleft centres between 1985 and 2000 on Turkish populations (16). Their findings are comparable to our Bangladeshi and Pakistani outcome, keeping in mind that the surgeons involved in the treatment of UCLP patients still practise the same protocols of surgery in such populations. Eighty percent of the UCLP patients were fair to poor outcomes (GOSLON 3 and 4) in a Japanese population (9) while a study of 66 UCLP cases in Western Australia demonstrated a total GOSLON score of 3.17 (29).

These differences might be for the disparities in different techniques of cheiloplasty and palatoplasty and/or the experience of the surgeons. The current findings of the study demonstrated that the treatment outcome in the three populations was comparable. The Malaysian subjects presented comparatively favourable outcomes than the other two populations and Bangladeshi subjects tended more toward unfavourable outcomes. It should be noted that presenting different outcomes in different populations and races of treatment outcomes based on the DAR seemed to be attributable to surgical procedures, but the racial difference in the craniofacial morphology also deserves consideration.

### Effects of Multiple Factors on DAR

The present study explored the factors that may be associated with the unfavourable DAR in three populations. Gender (male/female), side of cleft (left/right), type of cleft (complete/incomplete), the presence of a family history of cleft and class III malocclusion in the family, palatoplasty, and cheiloplasty were the independent variables. Crude logistic regression analysis was used to assess the association between each factor and DAR.

In this study, we found more males (59, 52, and 52% in Malaysia, Bangladeshi and Pakistani, respectively) than females. The results correspond to the outcomes with the previous studies (1, 12). Moreover, male UCLP subjects were significantly associated with unfavourable DAR in the Bangladeshi population though Malaysian and Pakistani UCLP subjects did not show any significant associations. Contrariwise, Arshad et al. (12) reported female gender had more unfavourable DAR in Pakistani UCLP subjects previously. Yet the actual cause of this phenomenon is still unconvinced (30).

All the subjects in the present study were between 5 and 12 years old. Fifty two percent of subjects were in the early mixed dentition period in the Malaysian population and 52 and 61% were in Bangladeshi and Pakistani populations, respectively. Left-sided clefts were observed more in all the populations. The prevalence of the left-sided cleft were 57, 52, and 63% in Malaysia, Bangladesh, and Pakistan, respectively. The higher prevalence of left-sided cleft cases than the right side was also reported in the literature (13). The majority of Malaysian (70%) and Bangladeshi (61%) subjects consist of the incomplete type of UCLP while all the subjects from Pakistan were complete UCLP. Anatomically, when a failure of fusion occurred between the hard and soft tissue structures of the soft palate, hard palate, alveolus, and lip refers to the complete type of UCLP. Habitually complete type of UCLP treatment is quite complex rather than incomplete UCLP. However, this variable was not a statistically significant factor. Alam et al. (31) reported no significant association between age, side, and type of UCLP in a recent Japanese UCLP study which is constant with our findings.

The choice of different techniques of cheiloplasty depends on the surgeon's preferences and different cases as well. Sometimes cheiloplasty is carried out alone or sometimes goes together with primary palatoplasty. However, noticeable developmental retardation was reported when the performances are completed together (32). In this study, the Millard technique was found to be common for lip surgery in Malaysian subjects while the modified Millard technique was more common in Bangladeshi and Pakistani UCLP subjects.

Millard technique of cheiloplasty significantly showed a favourable outcome of DAR than the modified Millard technique in Bangladeshi subjects. Adetayo et al. (2) compared the Millard and Tennison–Randall's techniques of cheiloplasty among Nigerian UCLP subjects and found no significant differences. Both techniques showed a favourable outcome of DAR. Two types of modified Millard techniques were performed in Japanese UCLP subjects where Modified Millard with vomer flap was significantly associated with unfavourable DAR than modified Millard only (17). Due to the rotation advancement methods of the modified Millard technique, the development of tension is attributed which tends toward unfavourable growth patterns (33). Kuijpers-Jagtman and Long, (34) stated that the greater lip tension is anticipated to cause dentoalveolar contraction more willingly than skeletal changes. Nevertheless, the skeletal changes comprising an anterior portion of the maxilla in anteroposterior and transverse dimension has also been reported (35–38). However, lip length was not considered in the present study, which could justify the use of a modified Millard technique. Till to date, it is still doubtful which surgical technique provides the best outcome either for lip repair or for palate repair. The aim of the surgery, differences in the severity of the cases, the surgeon's experience, expertise, and preferences may affect the outcome of the surgery as well.

The primary aim of palatoplasty is to restore function and phonetics. Common traditional surgery techniques like von Langenbeck, Bardach technique, V-Y pushback were used to achieve these goals. In this study, all the subjects were treated with either the Bardach technique or von Langenbeck technique palatoplasty. Bardach techniques of palatoplasty was identified as a factor that resulted in unfavourable DAR among all populations.

The unfavourable effect of palatoplasty on speech, maxillary growth, upper dental arch, and dental occlusion has been extensively documented. Consistent comparative results of different methods are seldom documented. Abundant confounding factors i.e., the defect size, extension of the defect, time of surgery and prominently the growth response makes valuation difficult (3).

An earlier study reported that minor scar formation was the main cause of the better outcomes of the von Langenbeck technique (39). Yet some researchers concluded their studies with no significant difference in the outcomes of different surgical techniques (40, 41). Sato et al. (42) found favourable outcomes among Brazilian UCLP subjects who had used the von Langenbeck technique of palatoplasty. Its favourable effects on outcomes have also been discussed in the previous study (43).

In contrast, the Bardach technique forms scar which would be responsible for the growth restriction. Fistula formation has also been associated as a drawback of this technique when performed to repair larger defects (44). However, Rossell-Perry et al. (45), reported no significant differences between twoflap (Bardach technique) and oneflap palatoplasty on DAR. Moreover, the patient treated with the Bardach technique achieved more normal speech (46). Altered surgical methods are employed to determine the outcome on DAR, and it can be stated from the studies that the treatment outcome of UCLP subjects is influenced by the surgical technique used.

Because of the disagreement between the outcomes of the studies, it is recommended that the correlation between treatment outcome (DAR) and the effect of multiple factors be better explored.

The present study provided information that postnatal treatment factors are associated with favourable and unfavourable DAR in all three populations. These findings could warrant a modification of management protocols to ensure improvement in future cleft outcomes.

The present study has achieved its aim by getting precise informative findings however it has some unavoidable limitations. Obtaining data from a single centre was one of the limitations of this study therefore the findings may not be generalizable. A multi-centre and prospective study design may provide more insights of the attributes of the variability of subjects.

## CONCLUSION

The present study revealed that,

### Malaysian Population

1. The mean GOSLON score was 2.97
2. von Langenbeck technique of palatoplasty significantly associated with favourable DAR among Malaysian UCLP children.

### Bangladeshi Population

1. The mean GOSLON score was 3.40
2. Female subjects, Millard technique of cheiloplasty and von Langenbeck technique of palatoplasty significantly associated with favourable DAR among Bangladeshi UCLP children.

### Pakistani Population

1. The mean GOSLON score was 3.09
2. von Langenbeck technique of palatoplasty significantly associated with favourable DAR among Pakistani UCLP children.

## Data Availability Statement

The original contributions generated for this study are included in the article/supplementary material, further inquiries can be directed to the corresponding author/s.

## Ethics Statement

The studies involving human participants were reviewed and approved by the study was approved by the Ethics Committee of the Hospital Universiti Sains Malaysia (HUSM) [USM/JEPeM/17100564]. Written informed consent to participate in this study was provided by the participants' legal guardian/next of kin.

## Author Contributions

SH, MK, and MA designed the study, performed the data collection, data analysis and interpretation, wrote the manuscript and reviewed the manuscript. SH, MK, MA, and WW performed the data collection, data analysis and interpretation, and wrote the manuscript. All authors declare that they contributed to critical review of intellectual content and approval of the final version to be published.

## Conflict of Interest

The authors declare that the research was conducted in the absence of any commercial or financial relationships that could be construed as a potential conflict of interest.
